# Molecular docking analysis of VEGF with compounds from tomato

**DOI:** 10.6026/97320630018478

**Published:** 2022-05-31

**Authors:** Madhan Krishnan, Shyamaladevi Babu, Suni Ann Thomas, Jaya Senthilnathan Surulivel, Kavitha Ayyanar

**Affiliations:** 1 Faculty of Allied Health Sciences, Chettinad Hospital and Research Institute, Chettinad Academy of Research and Education, chengalpattu District, Tamilnadu, India; 2 Department of Biochemistry, Al Azhar Medical College Thodupuzha, Kumaramangalam, Kerala, India; 3 Department of General Medicine, Vels Medical College & Hospital, Vels Institute of Science Technology & Advance Studies (VISTAS), Chennai, Tamilnadu, India; 4 Department of Biochemistry, Vels Medical College & Hospital, Vels Institute of Science Technology & Advance Studies (VISTAS), Chennai, Tamilnadu, India

**Keywords:** Molecular docking, VEGF, lung cancer, tomato

## Abstract

Vascular endothelial growth factor (VEGF) is linked with Non-small cell lung carcinoma (NSCLC). Therefore it is of interest to document data on the molecular docking analysis of VEGF with compounds from tomato for consideration drug discovery. Data shows that compounds Kaempferol-3-O, Quercetin, Naringenin & Rutin show optimal binding.

## Background:

Lung cancer is a serious threat to human health. This condition has been the world's most dangerous malignancy [1]. Every year, around 1.6 million new cases of lung cancer are identified worldwide. Lung cancer has the highest mortality rate among malignant tumours [2]. Histological subtypes of lung cancer are primarily classified into non-small cell lung cancer (NSCLC) and small cell lung cancer (SCLC), accounting for between 85 percent and 15 percent of patients with lung cancer. The 5-year survival rate of initial NSCLC patients is approximately 40% following surgery [3]. Even then, about 70 % of patients had localized or distant metastases at the point of surgery [4]. During the meantime, SCLC is more susceptible to radiotherapy and chemotherapy relative to NSCLC. There are indeed limitations for the clinical care of SCLC. For instance, SCLC is vulnerable to drug resistance [5]. Factors and signaling pathways associated with tumor angiogenesis have been identified as promising targets for therapeutic strategies in different tumor types, including lung cancer [6]. Vascular endothelial growth factor (VEGF) A (VEGF) and VEGF receptor (VEGFR) play a key role in angiogenesis, facilitating endothelial cell proliferation, migration and invasion [7]. VEGF increases the vascular permeability of established vessels, increasing the extravagance of plasma proteins that provide a provisional scaffold for activated endothelial cells to migrate. In addition, VEGF enhances the homing of bone marrow vascular precursor cells [8]. Recent research indicates that VEGF specifically targets tumor cells that lead to tumor progression and metastases [9]. VEGF excess expression and/or high serum VEGF levels have been documented for both NSCLC and SCLC [10]. The expression of VEGF transcript and protein was identified in several human NSCLC cell lines [11]. The levels of VEGF protein differed across cell lines. Various studies have documented that VEGFRs were expressed and activated in NSCLC cell lines suggesting that autocrine loops could be involved in these cells. The expression of VEGF and phosphorylate VEGFR2 has been found in different SCLC cell lines, indicating that VEGF may preserve cellular functions in SCLC through autocrine mechanisms [12]. Therefore it is of interest to document data on the molecular docking analysis of VEGF with compounds from tomato for consideration drug discovery.

## Materials and Methods:

### Protein preparation:

The X-ray crystal structure of VEGF (PDB code: 1FLT) was downloaded from the Brookhaven Protein Data Bank (www.rcsb.org/pdb).

### Ligand preparation:

10 compounds (Table 1 - see PDF) known from the tomato plant were collected from the literature. The structures of these compounds were dowmloaded from the PubChem Compound Database in the Spatial Data File (SDF) format and translated to the PDB file format by using Online Smile Translator. Energy minimization of ligands was completed using Open Babel software with a steepest 139 descent using uniform force fields and then translated to PDBQT format.

### Molecular docking:

AutoDock (V. 4.0) could be used in the PyRx GUI to validate the binding capability of the selected ligands to the selected target [13]. The grid configuration report was created using the Pyrex Auto Grid software. Execution was also used to know / predict amino acids that come into contact with ligands at the active protein site. Results less than 1.0Å in the position root-mean-quarter deviation (RMSD) were considered to be optimal and grouped together to find an acceptable binding. The highest binding energy (most negative) is considered as a ligand with high binding affinity. The docking poses collected for each compound have been rated according to their dock score feature and the best docking result was further analyzed using.

## Results and Discussion:

In order to recognize the interaction of the selected compounds with the active VEGF site, the compounds have been submitted to molecular docking simulation studies performed using the PyRx docking method using the Autodock VINA program. The molecular docking of bioactive compound at the VEGF binding site was carried out on the basis of the reported VEGF (PDB code: 1FLT) and complex structures, and the highest suitable binding modes of the best compounds were shown in Figure 1(see PDF). The binding energy and H-bond information have been shown in Table 2(see PDF), where the created docked complexes have been examined on the basis of binding affinity values (kcal/mol) and bonding interaction patterns (hydrogen, hydrophobic, and electrostatic). Compounds have strong hydrogen bonding interactions through amino acid residues GLN-22, THR-31, LEU-32, GLN-37, CYS-57, GLY-59, ASN-100 and CYS-102. By using pymol, the docking results were then compared to see the interaction produced the best binding energy, out of 10 dockings performed. Many of the compounds showed very strong interactions with the VEGF receptor. We sorted the four compounds on the basis of the binding energy. The strongest docking interaction between VEGF and four compounds was shown in Figure 1(see PDF), with binding energy ranging from-7.2 to 5.9 kcal /mol. Analysis of the docking results showed that selected compounds interact with VEGF protein via H bond interactions. These compounds showed the strong interaction with active site residues. The presence of the H-bond interactions enabled the complex to attain the specified configuration of the complex structure. The selected four compounds showed very close interactions with the VEGF receptor [14]. As per Daisy et al. Kaempferol-3-O, Kaempferol-3-O, Naringenin & Rutin form an H bond with the VEGF receptor and their duration is also below 3A0. Data shows that tomato plant compounds have potential anti lung cancer activity.

## Conclusion:

Data shows that compounds Kaempferol-3-O, Quercetin, Naringenin & Rutin show optimal binding with VEGF.
